# Studying the Genetics of Resistance to CyHV-3 Disease Using Introgression from Feral to Cultured Common Carp Strains

**DOI:** 10.3389/fgene.2017.00024

**Published:** 2017-03-10

**Authors:** Roni Tadmor-Levi, Efrat Asoulin, Gideon Hulata, Lior David

**Affiliations:** ^1^Department of Animal Sciences, Robert H. Smith Faculty of Agriculture, Food and Environment, The Hebrew University of JerusalemRehovot, Israel; ^2^Institute of Animal Science, Agricultural Research Organization, Volcani CenterRishon LeZion, Israel

**Keywords:** Koi herpes virus, family selection, backcross, cohabitation, disease model, *Cyprinus carpio*, infectious disease, fish breeding

## Abstract

Sustainability and further development of aquaculture production are constantly challenged by outbreaks of fish diseases, which are difficult to prevent or control. Developing fish strains that are genetically resistant to a disease is a cost-effective and a sustainable solution to address this challenge. To do so, heritable genetic variation in disease resistance should be identified and combined together with other desirable production traits. Aquaculture of common carp has suffered substantial losses from the infectious disease caused by the cyprinid herpes virus type 3 (CyHV-3) virus and the global spread of outbreaks indicates that many cultured strains are susceptible. In this research, CyHV-3 resistance from the feral strain “Amur Sassan” was successfully introgressed into two susceptible cultured strains up to the first backcross (BC_1_) generation. Variation in resistance of families from F_1_ and BC_1_ generations was significantly greater compared to that among families of any of the susceptible parental lines, a good starting point for a family selection program. Considerable additive genetic variation was found for CyHV-3 resistance. This phenotype was transferable between generations with contributions to resistance from both the resistant feral and the susceptible cultured strains. Reduced scale coverage (mirror phenotype) is desirable and common in cultured strains, but so far, cultured mirror carp strains were found to be susceptible. Here, using BC_1_ families ranging from susceptible to resistant, no differences in resistance levels between fully scaled and mirror full-sib groups were found, indicating that CyHV-3 resistance was successfully combined with the desirable mirror phenotype. In addition, the CyHV-3 viral load in tissues throughout the infection of susceptible and resistant fish was followed. Although resistant fish get infected, viral loads in tissues of these fish are significantly lesser than in those of susceptible fish, allowing them to survive the disease. Taken together, in this study we have laid the foundation for breeding CyHV-3-resistant strains and started to address the mechanisms underlying the phenotypic differences in resistance to this disease.

## Introduction

Securing the supply of healthy and nutritious food is a significant challenge for humanity. It is the major one for agriculture now, and will be more so in the future. Aquaculture, as an important food supply sector, is facing several major challenges on the way to achieve sustainable production and maintain its steady growth. One such challenge is how to overcome the serious problems caused by a whole range of infectious diseases that spread and inflict considerable damages on this industry, and to some extent also on the adjacent natural habitats (Murray and Peeler, 2005; Pulkkinen et al., 2010; Johansen et al., 2011). Furthermore, since aquaculture is a general term for culturing numerous species using different rearing methods across different conditions and geographic areas, it suffers from numerous diseases that emerge and outbreak according to these same variables (Walker and Winton, 2010; Lafferty et al., 2015). Since measures to prevent and control disease outbreaks are more limited for fish than for land farm animals, disease resistance, as a sustainable solution to this problem, is highly sought for (Chevassus and Dorson, 1990; Fjalestad et al., 1993; Stear et al., 2012).

Similarly to humans and land animals, few lines of action are practiced to battle disease outbreaks in fish. Although treatments can be applied under specific conditions to some diseases, prevention is preferable, especially when considering the safety, economical, and practical aspects of applying medicines in aquaculture. Usually, vaccine development provides a faster on-site solution to infectious diseases (Sommerset et al., 2005; Gudding and van Muiswinkel, 2013), whereas development of genetically disease resistant lines takes longer but has its advantages in terms of sustainability. Therefore, combining all strategies to battle fish diseases is highly important to achieve a long-term development of sustainable industry.

One such infectious disease is caused by the cyprinid herpes virus type 3 (CyHV-3), also called Koi herpes virus (KHV), which belongs to the double-stranded DNA *Alloherpesviridae* family. While the presence of this virus has been detected in several cyprinid species, the disease is specific to the common carp (*Cyprinus carpio*), affecting both cultured food strains and ornamental Koi varieties. Reports on outbreaks of this disease started in the 1990s and since then, the disease had spread to almost all places in which common carp is cultured or kept (Ilouze et al., 2011; Rakus et al., 2013; Adamek et al., 2014). Since common carp is among the top 5 species produced in aquaculture and since outbreaks of this disease can lead to significant losses of 50–100% of the fish, CyHV-3 resistance is a desirable trait for inclusion in breeding programs. A few studies so far have looked at survival levels of different strains following infection by CyHV-3 (Shapira et al., 2005; Dixon et al., 2009; Ødegård et al., 2010; Piačková et al., 2013; Ito et al., 2014). Only a subset of these studies included F_1_ crosses between strains in their analysis. Differences that were found among strains and among F_1_ families, as well as similarity between parents and their F_1_ progeny were all good indicators for the presence of a genetic component controlling CyHV-3 resistance. However, despite the need and the feasibility for success, results of breeding for CyHV-3 resistance have not been reported yet.

For a breeding program to succeed, it should start from a population with a considerable amount of heritable trait variation. In this study, variation in CyHV-3 resistance was studied in strains, families, and generations. A set of first backcross (BC_1_) families was established and analyzing the wide trait variation found among these families indicated a substantial component of additive genetic variation. Furthermore, understanding the resistance mechanism is important and in this respect, the differences in resistance between susceptible and resistant fish were studied by testing the protective effect of scale coverage and by following the viral infection loads in tissues over time. Our results prove the feasibility of breeding for CyHV-3 resistance and open up the way to understand the differences between susceptible and resistant fish. Both the practical and basic aspects of this study are imperative for solving the problems caused by CyHV-3 and for better understanding of fish immunogenetics.

## Materials and Methods

### Fish Strains and Families

**FIGURE 1:**
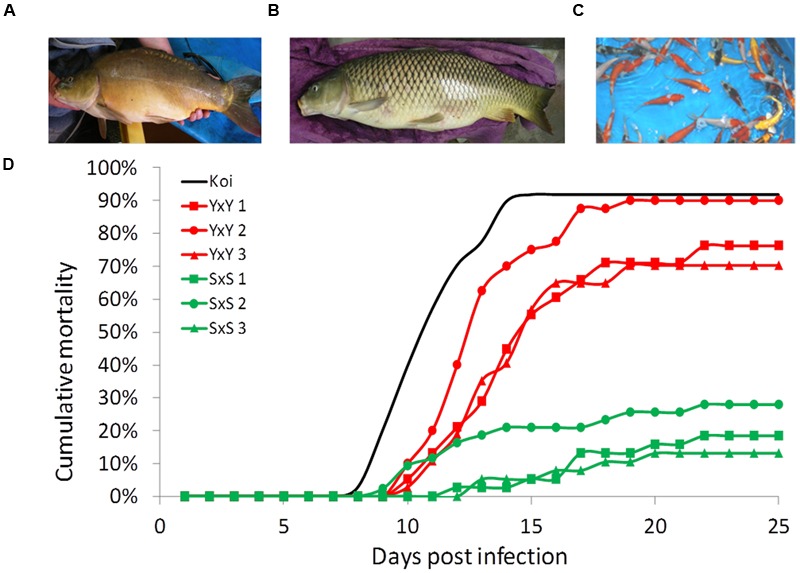
**Strain representation and typical cumulative mortality curves. (A)** The cultured strain Yugoslavian Našice (“Y”) with mirror phenotype and round body shape. **(B)** The feral strain Amur Sassan (“S”) with full-scale coverage and torpedo shaped body. **(C)** Group of ornamental carp “Koi.” **(D)** Cumulative mortality curves in cohabitation challenges of representative susceptible “YxY” and resistant “SxS” families together with the average curve for the “Koi” control groups.

Four strains of common carp (*C. carpio*) were used in this study. Two strains of cultured edible carp—Yugoslavian (“Y”) (Našice; introduced to Israel from former Yugoslavia in the 1970s) and Dor-70 (“D”) (bred in Israel; Wohlfarth et al., 1975). These strains have favorable agricultural traits such as reduced scale-coverage (mirror phenotype) and a high height/length ratio (**Figure 1A**). In Israel, these two strains are used as parental lines and their cross progeny are reared as the commercial edible fish. In addition, one feral strain—Sassan (“S”) [Amur Sassan; imported to Israel from the Czech republic, originally from the former USSR (Ukraine); Pokorný et al., 1995; Shapira et al., 2005] that presents unfavorable phenotypes for aquaculture, such as full-scale coverage and elongated body shape (**Figure 1B**). Finally, “Koi” fish groups of mixed varieties were used as controls (**Figure 1C**). Most näive “Koi” fish were obtained at ∼10 g weight from Kibbutz Gan Shmuel and Kibbutz Ma’agan Michael fish breeding centers in Israel. All families of other carp strains were produced and reared as described by Bar et al. (2013), in our fish facility at the Robert H. Smith Faculty of Agriculture, Food and Environment of the Hebrew University of Jerusalem in Rehovot, Israel.

Over 6 years, 72 parent fish (**Table 1**) were used to produce 81 families: 27 families from the three carp strains, 27 F_1_ families of crosses among the three carp strains, and 27 BC_1_ families of F_1_ fish backcrossed to the parental commercial strains. For some of the families, the same F_1_ or the cultured strain parent was used, creating half-sib families (**Table 2**). All these 81 carp families were challenged to study their level of resistance to CyHV-3. In each year, multiple families were tested in replicates, therefore the duration of all the experiments in each year was long. This resulted in age and size variation between experiments of each year, since fish groups were continuously cultured in parallel to the experiments. In addition to challenge experiments, for some of these families tissues were sampled for DNA extraction.

**Table 1:** Number of parents used for crosses by year, strain, and sex.

	“Y”		“D”		“S”		“SxD”		“SxY”		Total	
	F	M	F	M	F	M	F	M	F	M	F	M
2010	4	4	3	1	4	4					11	9
2012	1	3	2	3	2	3					5	9
2014	2	6	2	1	1	3		6		4	5	20
2015	1	3			1	2					2	5
2016	2	4			1	1			6		9	5
Total (unique)^∗^	10 (9)	20 (17)	7	5	9 (7)	13 (11)		6	6	4	32 (29)	48 (43)

*^∗^In some cases the same parents were used for crosses in more than 1 year, so numbers of unique fish are reported.*

**Table 2:** Number of families that were challenged by year and cross-type.

	SxS	YxY	DxY	DxD	SxY	SxD	Yx (SxY)	Dx (SxD)	Total
2010	4	3	3	1	3	2			16
2012	1		2		5	6			14
2014	3	6		1	3		4	12	29
2015	2	3			3				8
2016	1	2					11		14
Total	11	14	5	2	14	8	15	12	81

### CyHV-3 Challenge Trials

Once fish families reached an average weight of 10–30 g and age of 3–12 months, they were challenged with wild-type CyHV-3 infection by cohabitation with previously infected fish (shedders), to determine their survival levels. To assure a successful infection by cohabitation, infection was initiated on non-experimental groups. Initially, 30–50 naïve “Koi” fish were injected intraperitoneally with 100 μL of 10 p.f.u/μL WT CyHV-3 virus (kindly provided by KoVax Ltd., Israel) and placed in tanks containing 200–300 L with water temperature kept at 21–24°C. To establish infection by cohabitation, every 4–6 days, more naïve “Koi” fish were added to the tank and mortalities were recorded daily. Typical mortalities from CyHV-3 infection started at days 8–12 post-infection. When groups of cohabitated “Koi” fish started typical mortalities, challenges of experimental families were initiated by adding them to the tank with the “Koi” shedders.

For each tested family, two to four replicated challenges were performed (except in 2010 when only one replicate was performed per family) on a sample of 10–60 fish per replicate [average of 28.8 fish and standard deviation (SD) of 12.36, depending on family size]. In each challenge experiment, two to six families per tank were added, each marked by clipping different fins. Prior to challenge, fish were given formaldehyde or potassium permanganate treatments as a preventative measure to reduce the risk of biased mortalities due to ectoparasites infection. Families were randomly assigned to tanks, so that each replicate of a family shared the experimental tank with different families each time, to eliminate possible effects of families on each other and to eliminate effect of size and age. As a positive control for each challenge experiment, a susceptible group of fish was used, usually from the highly susceptible “Koi,” but sometimes from known susceptible carp families. Tanks holding the naïve fish, from which the test groups were sampled, served as negative controls to verify no other mortalities were observed during the experiment. Water quality was monitored during the experiments to ensure no further stress on the fish from high levels of chlorine, ammonia or nitrite. If needed, freshwater were added. Typical experiment lasted for 20 days, during which dead fish were counted daily for each family and removed till mortalities ceased. At the end of an experiment, remaining fish for each family were counted. Virus DNA presence in kidney and intestine of dead fish was routinely verified by polymerase chain reaction (PCR).

### Tissue Sampling Throughout Infection

For quantification of viral DNA levels throughout infection, 32 fish from a resistant background group (“SxS”) and 76 fish from a susceptible background group (“YxY”) were exposed to CyHV-3 infection by cohabitation (as described above). On day 0 (pre-infection) and on days 4, 6, and 8 post-infection, six fish from each group were anesthetized with 2-phenoxyethanol and euthanized. From each fish, samples of spleen, kidney, foregut, hindgut, skin, gills, brain, and liver were taken, placed in tubes containing 100% ethanol and stored at -20°C. The excess fish from both groups served as a control for charting mortality curves and estimating the actual survival level of each group.

### DNA Extraction

DNA was extracted using modified protein salting-out method (Turtinen and Juran, 1998). In brief, approximately 5 mg of tissue sample was dried from ethanol and placed in 550 μL of cell lysis solution [50 mM Tris–HCl, pH 8.0, 50 mM ethylenediaminetetraacetic acid (EDTA), 100 mM NaCl]. Sodium dodecyl sulfate (SDS) solution was added to a concentration of 1% and cells were further lysed by incubation with 1 μL of proteinase K solution (20 mg/mL) for 2 h at 50°C. The lysate was supplemented with 300 μL of 5 M NaCl, vortexed and centrifuged (13,000 rpm, 10 min) to precipitate the proteins. The supernatant liquid phase was mixed with 900 μL of freezer cold 100% isopropanol, incubated for 2 h at -20°C, and centrifuged (13,000 rpm, 5 min). The resulting DNA pellet was washed with 700 μL of 70% ethanol, dried for 15 min and dissolved overnight in 100 μL of double distilled water at 4°C. DNA concentration and quality (optical density, OD_260_/OD_280_ ratio) were measured using NanoDrop ND-1000 (NanoDrop Technologies) and visually examined by 1.5% Tris/Borate/EDTA (TBE) agarose gel electrophoresis. DNA samples were stored at -20°C until used for further analysis.

### PCR for Viral DNA Detection

Viral DNA presence in dead fish was verified using PCR. Primers designed for the viral gene *major capsid protein* (*MCP*), developed by Dishon et al. (2007) (MCP-F: 5′-GGATCCCCAGGCGTACTTCATGTCCT-3′ and MCP-R 5′-GGATCCACGATGGGCACCAACTTTAG-3′), were used to detect the presence of viral DNA. As an endogenous PCR control, primers designed for the mitochondrial gene *cytochrome oxidase I* (*COX I*) developed by Ward et al. (2005) (cox1a_fw: 5′-TCAACCACCCACAAAGACATTGGCAC-3′ and cox1a_rv: 5′-TAGACTTCTGGGTGGCCAAAGAATCA-3′) were used. For PCR amplification, 1 μL of undiluted DNA sample (30–150 ng/μL) was added to a mix containing 2 μL of 10X Taq buffer, MgCl_2_ (25 mM), dNTPs (6.6 mM of each), Taq polymerase (1.5 U), primer mix (forward + reverse, 2.5 μM of each) and water to reach a total volume of 20 μL per sample. The PCR profile used was 94°C for 2 min, followed by 35 cycles of 94°C for 10 s, 60°C for 1 min, and 72°C for 1 min, and 72°C for 10 min for final elongation. The presence of viral DNA was analyzed by electrophoresis of the PCR products on a 1.5% TBE agarose gel stained with ethidium bromide. PCR products were visualized using Gel Doc XR+ (BIO-RAD) and using ImageLab software (BIO-RAD). For each PCR analysis, DNA from a known CyHV-3 infected fish and a naïve fish were used as positive and negative controls, respectively, as well as one water sample (no DNA template negative control).

### qPCR for Measuring Viral DNA Levels

Relative viral load of CyHV-3 was measured by real-time quantitative PCR. Primers for *elongation factor 1-alpha* (*EF1α*), reference carp gene were designed based on *C. carpio* EST libraries from National Center for Biotechnology Information (NCBI) by Bar et al. (2013) (EF1a_F: 5′-CAAGGTCACGAAGTCTGCAC-3′ and EF1a_R: 5′-CACGAGGTTGGGAAGAACAT-3′). Primers for measuring the viral load of CyHV-3 were designed by Dishon et al. (2007) to amplify a 200-bp amplicon of the CyHV-3 *B22R homolog*-NHRT (NHRT-F: 5′-CCAGATCCACCAGCTGCTGT-3′ and NHRT-R: 5′-AAGATGGGATCTCTCGGAGG-3′). Calibration curves were generated for each primer pair to calculate amplification efficiency and the preferred concentration of DNA template to use. To generate calibration curves for both primer pairs, a known CyHV-3 positive sample was used for seven serial dilutions of 2× each, starting with a concentration of 200 ng/μL.

For the PCR, a master mix containing 6 μL water, 2 μL of primer mix (forward + reverse, 2 μM each) and 10 μL Platinum SYBR Green qPCR SuperMix-UDG (Invitrogen) per sample was prepared. From this master mix, 18 μL were placed into each well of a 96-well plate and complemented with 2 μL of the DNA sample (50 ng/μL). The plate was centrifuged and placed in the LightCycler^®^ 96 (Roche). The following PCR protocol was used: pre-amplification hot start segment (50°C for 2 min, 95°C for 2 min), amplification segment (95°C for 15 s, 60°C for 30 s, and 72°C for 15 s with a single fluorescence measurement), repeated for 40 cycles and a dissociation curve segment (95°C for 10 s, 65°C for 30 s, and ramp up to 97°C, with increment of 0.1°C per/s and continuous fluorescence measurement). Relative concentration (Cq values) were measured at fluorescence threshold of 0.2 with LightCycler^®^ 96 software version 1.1.0.1320 (Roche) and reported values were used as the data for further analysis.

### Survival Data Analysis

For each family replicate, the survival level was calculated as the proportion of dead fish from total fish (dead + survived). Another measure, “mortality pattern,” was calculated for testing if some groups died earlier than others. This measure was calculated as the ratio between the number of days since the beginning of mortalities until 50% of mortalities were reached (e.g., if during the experiment a total of 20 fish died, the number of days that have passed since the first fish has died until the 10th fish has) and the whole duration of mortality (i.e., the number of days that passed since the first fish has died until the last fish has). Both for survival level and for “mortality pattern” the final value for each family was the mean of all replicates. These proportional values were arcsine-transformed [Y′ = Arcsine (√Y)] to obtain normally distributed values and equal variances (Shapiro–Wilk test was used to test if data is of normal distribution; Bartlett’s and Levene’s tests were used to test whether variances were equal). Each of these sets of transformed values was then used separately in the different statistical analyses. Since experiments were held in different tanks and in different years, the effects of these factors were tested and found insignificant for both measures (data not shown). Therefore, it was not necessary to correct the data for tank or year effects.

One-way analysis of variance (ANOVA) was used to analyze the effect of different strains or different parents, separately on each of two variables, survival level and “mortality pattern”. Tukey–Kramer honestly significant difference (HSD) test was used to compare means *post hoc*. A *t*-test was used to compare means of scaled and mirror fish groups. χ^2^ tests were used for comparing survival levels between scaled and mirror fish in each of the BC_1_ families. For the χ^2^ tests, individuals were categorized into one of four combinations of died/survived–scaled/mirror and compared. To compare the variation between cultured, F_1_ and BC_1_ backgrounds an *F*-test was used (*P*-values were adjusted to multiple comparisons by a Bonferroni correction). All statistical analyses were done using the JMP12 software (SAS Institute, Cary, NC, USA).

### Genetic Variation Estimates

Our data structure did not allow traditional heritability estimates because it was not balanced enough and often contained first or second generation sibs as parents. Therefore, other analyses were performed in order to qualitatively estimate the variation components of the CyHV-3 resistance phenotype. First, variation among means of BC_1_ families of different parents was compared to variation between families within each parent. Similarity within parents compared to variation between parents would indicate the presence of an additive genetic variation component. In a second analysis to find evidence for an additive genetic component, two basic additive inheritance assumptions were used: first, that the progeny average is a good estimate for the parent’s value and second, that a progeny value should approximate the average of its parents. Each parent was assigned with the average value of its progeny. Using the value for each parent, the expected value of the progeny was calculated as the average of its parents. Significant correlation between the expected and the observed values would indicate the presence of an additive genetic variation component.

### Relative Viral Load Data Analysis

Since sample measurements were done across several PCR plates, common samples were added to each plate and their measured Cq values were used for normalizing between experiments done on different days and plates. Cq values of each sample were measured in two to four technical replicates and their average was used for further analysis. Relative viral load was calculated using the “–ΔΔCp with efficiency correction” calculation method (Pfaffl, 2001). The Cq values of *NHRT* viral gene in samples were normalized to the carp gene *EF1α* and calculated relative to its mean level in spleen, liver, and kidney of resistant fish at day 0. Relative concentration values were presented as fold changes but for statistical analysis, values were Log_10_-transformed to obtain normally distributed values and equal variances.

A three-way ANOVA model was used to test for the effect of three factors “day” (0, 4, 6, or 8), “tissue” (spleen, kidney, and liver) and “phenotype” (resistant or susceptible) and all the possible interactions between them. A *t*-test was used to compare resistant and susceptible fish within each “day” as well as resistant and susceptible fish within each “tissue.” Dunnett’s test was used to compare each of days 4, 6, and 8 to control day 0 within each phenotype. Tukey–Kramer test was used to compare among means of different tissues, separately in resistant and susceptible fish. All statistical analyses were done using the JMP12 software (SAS Institute, Cary, NC, USA).

### Ethics Statement

Protocols for fish reproduction, rearing, sampling, and dissection were reviewed, approved, and granted with permission numbers AG-07-11077-5 and AG-11-13059-5 from the Animal Research Ethics Committee of the Hebrew University of Jerusalem. All experimental procedures followed these approved protocols in accordance with the recommendations of the review committee and in accordance with national laws and regulations for animal research ethics. Because of the nature of this research and the large variability in the resistance/susceptibility levels found among fish families, power calculations were made with regards to the number of fish used in the experiments and the amount was reduced to the minimum necessary for reaching results that would significantly (*P* < 0.05) differentiate two groups with 10% difference in survival level. Although disease symptoms can be seen prior to death, these symptoms were not predictive of mortality or survival. From mild signs like loss of appetite and up to external lesions could be seen also in resistant and surviving fish, while susceptible fish die quickly after external lesions occur. Therefore, we could not find a reliable endpoint to the challenge experiments other than counting daily mortalities. Soon after mortalities in the challenge experiments ceased, surviving fish were recovered if kept or anesthetized and euthanized if not, since survivors are immunized and cannot be used for further challenge experiments.

## Results

### Survival Levels of Strains and Crosses

**FIGURE 2:**
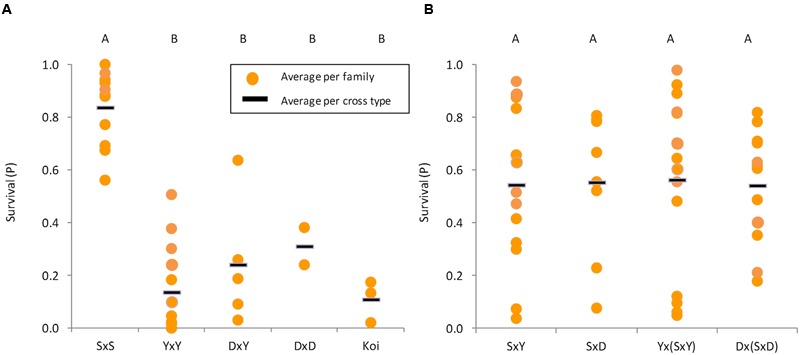
**Survival levels of different strains and cross types.** In both panels each yellow dot represents the average survival of a family and the black line represents the average per cross type. Cross types not sharing the same capital letters on top are significantly different (Tukey–Kramer test, *P* < 0.05). **(A)** Families belonging to parental strains (“S” is feral, while “Y,” “D,” and “Koi” are cultured). **(B)** Cross types between feral and cultured strains from F_1_ and BC_1_ generations.

Our disease challenge model was applied to test for the survival levels of crosses involving three common carp strains: Yugoslavian (“Y”), Dor-70 (“D”) and Amur Sassan (“S”) (**Figures 1A,B**). In line with previous reports, also our results confirmed that the cultured strains, both edible and ornamental, were more susceptible to CyHV-3, whereas the feral “S” strain was rather resistant (**Figure 1D**). Therefore, to study the trait variation, the analysis was extended to several families from each of the edible and feral carp strains as well as to different groups of “Koi” (**Figure 2A**). Mean survival levels were 13.7, 24.1, 31.1, and 10.9% for “YxY,” “DxY,” “DxD,” and “Koi,” respectively, with some variability among families (SD of 0.14, 0.16, 0.24, and 0.02 for “YxY,” “DxY,” “DxD,” and “Koi,” respectively). Thus, mean survival levels of these cultured strains were low and not significantly different from each other (Tukey–Kramer HSD, *P* > 0.05). On the other hand, families of the feral “S” strain were resistant with a mean survival of 83.7% (SD of 0.14), which was significantly greater from that of all the cultured strains (Tukey–Kramer HSD, *P* < 0.05).

While being attractively resistant, this feral strain is not suitable for aquaculture. Therefore, given the limited resistance seen in the cultured strains and lesser level of variation among their families, introgression of resistance from the feral strain “S” into the cultured edible strains “D” and “Y” was attempted. We challenged 21 F_1_ families (“SxY” and “SxD”) and 27 BC_1_ families [“Yx(SxY)” and “Dx(SxD)”]. Wide variation in survival levels among families was noted in F_1_ and BC_1_ families (**Figure 2B**). Some families were as susceptible as the cultured strains, whereas others were as resistant as the feral strain. The “SxY,” “SxD,” “Yx(SxY),” and “Dx(SxD)” had SD values of 0.29, 0.27, 0.33, and 0.21, respectively. Variation among families of all cultured strains together was significantly lesser than among F_1_ (*F*-test, *P* = 0.028) and BC_1_ (*F*-test, *P* = 0.022) families. Variation in F_1_ and BC_1_ families was similar (*F*-test, *P* = 0.996). Therefore, producing F_1_ and BC_1_ families generated wider variation in survival levels, which is important for further breeding. Furthermore, mean survival levels for “SxY,” “SxD,” “Yx(SxY),” and “Dx(SxD)” were 54.4, 55.3, 56.3, and 54.2%, respectively, and were not significantly different from each other (*F*-test, *P* = 0.999), indicating that resistance from the feral strain could be successfully inherited to subsequent generations.

Finally, since mortalities were counted daily, our data allowed analysis of “mortality pattern,” which is a measure to test if one group accumulated mortalities earlier than another. The measure is calculated as day to 50% mortality relative to day to final mortality. Although mortality levels of feral and cultured strains differed considerably (i.e., 83.7% survival for feral “S” strain compared to 13.7% survival for cultured “Y” strain), no significant differences in “mortality patterns” were found between these strain types (*t*-test, *P* = 0.5705).

### Testing for a Heritable Component in CyHV-3 Resistance

**FIGURE 3:**
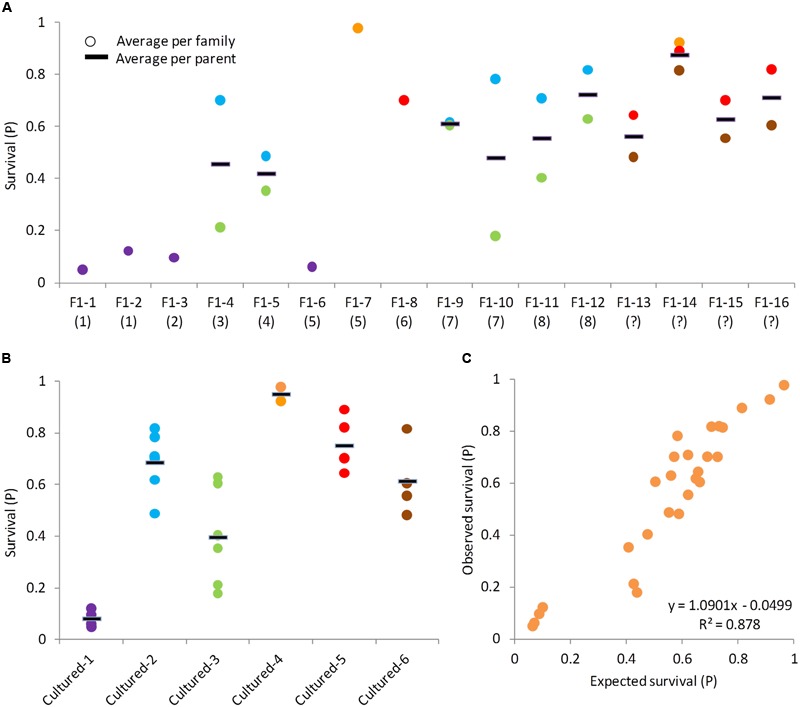
**Analyses of BC_1_ families indicating the additive genetic component in CyHV-3 resistance.** In all panels, each dot represents the average survival of a family and the black line represents the average per parent. **(A)** Survival levels of BC_1_ progeny by F_1_ parents. F_1_ parents are ordered from left to right by increasing survival levels of the families they came from (in parentheses is the survival level rank of the parent’s family). F_1_ parents coming from families with unknown survival level were labeled as (?) and placed on the right. Same dot color across F_1_ parents was given to families sharing the other, cultured parent. **(B)** Survival levels of BC_1_ progeny by cultured parents. Dot colors correspond to panel **(A)**. **(C)** Observed by expected survival level of BC_1_ families. Each dot represents the average of one family. Expected family survival was calculated as the average survival of its parents.

Since our data structure did not allow formal calculations of heritability estimates, alternative approaches were used to study the variation components of this heritable resistance (see description in the Section “Materials and Methods”). The F_1_ parents were ranked by increasing phenotypic value. A parent was ranked lower or higher based on the average survival level of the family it came from (when available). A corresponding trend in the average survival levels of their BC_1_ progeny was observed (**Figure 3A**). Progeny of susceptible parents were more susceptible and progeny of resistant parents were more resistant, indicating an additive genetic variation component. For the cultured parents, no family information was available, but we could assume they were rather susceptible based on our earlier estimates on these cultured strains (**Figure 2A**).

Besides the trend in averages, we also looked at the variances. For F_1_ parents that were crossed to more than one cultured counterpart, the variation among half-sib families was compared to the variation among means of F_1_ parents, and these were not significantly different (*F*-test, *P* = 0.3811). This could be because F_1_ parents were not randomly selected, as we generally preferred F_1_ parents that belonged to more resistant families, or perhaps due to dominant genetic effects. However, looking more carefully, we noticed that some F_1_ parents had half-sib BC_1_ progeny with similar levels of survival (e.g., F_1_-#14), while other with quite different survival levels (e.g., F_1_-#4). Therefore, the same test was done separately for the less variable and more variable F_1_ parents. The less variable F_1_ parents were F_1_-#5, F_1_-#9, F_1_-#12, F_1_-#13, F_1_-#14, F_1_-#15, and F_1_-#16 and for these, the variation among half-sib families was significantly lesser than the variation among F_1_ parents (*F*-test, *P* = 0.0204), indicating again an additive genetic component. Parents F_1_-#4, F_1_-#10, and F_1_-#11 were variable and for them the test result was not significant (*F*-test, *P* = 0.9527).

For the three more variable F_1_ parents, the differences between half-sib families could be explained by the effect of the other, cultured parents. The low-survival families of these F_1_ parents shared the same cultured parent (cultured-#3) and the high-survival families shared a different parent (cultured-#2), providing an explanation for this variability (**Figure 3B**). Variation among half-sib families of cultured parents is significantly lesser compared to variation among means of different parents (*F*-test, *P* < 0.0001). Therefore, also from the perspective of the other side of the susceptible parents’, evidence for additive genetic variation was found. While intuitively we expected the resistance to be inherited from the F_1_ parent, the more resistant BC_1_ families of the variable F_1_ parents suggested that cultured parents also contribute to resistance and have a significant contribution to parent–offspring similarity. Taken together, half-sib families were quite similar to each other in their mean survival and their survival level was determined by both parents as expected from a significant additive genetic component.

In theory, if there is a considerable additive genetic component to the observed trait variation, then the parent’s value can be estimated by its progeny value and the progeny value should approximate the mean of its parents’ values. Based on these assumptions, the expected survival level of the BC_1_ progeny were compared to the observed values and a significant correlation between the two was found (**Figure 3C**; *r* = 0.937, *P* < 0.0001), confirming the presence of a strong additive genetic variation component in this trait.

### Effect of Scale Pattern on Resistance

**FIGURE 4:**
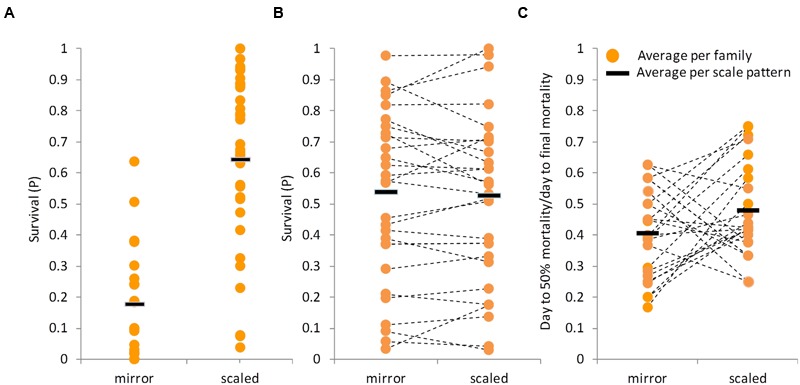
**Effect of scale pattern on survival level after CyHV-3 challenge.** In all panels, each dot represents the average survival of a family and the black line represents the average per scale pattern category. **(A)** Survival levels of families that did not segregate for scale pattern. Mirror category included families belonging to cultured strains, while scaled category families belonging to the feral strain and its F_1_ crosses. **(B)** Survival level by scale pattern in BC_1_ families segregating for scale pattern. Dashed lines connect between means of full-sibs groups with different scale pattern. **(C)** “Mortality pattern” by scale pattern in the segregating BC_1_ families. Dashed lines connect between means of full-sibs groups with different scale pattern.

Scale coverage, as part of the outer mucosal boundary, might confer some protection against the disease by reducing the infection efficiency. Not many fish species segregate for the presence of scale coverage (Kirpichnikov, 1981) and hence, offer the opportunity to test if scales have a protective role. As indicated before, the feral “S” strain has a full-scale coverage, while the cultured “D,” “Y,” and “DxY” strains have the mirror carp phenotype. Since the full-scale pattern is dominant, F_1_ fish are also fully scaled. However, in the BC_1_ progeny, the full-scale and mirror phenotypes segregated in a 1:1 ratio, as expected from a single gene trait. Since feral and F_1_ families were more resistant than “D,” “Y,” and “DxY” families, it could be that full-scale coverage confers some protection against the disease. To test this option, the mean survival between the two groups was compared: families with full-scale coverage and families with a mirror phenotype. Although variation among families with either phenotype was large, on average, fully scaled families had significantly greater survival level than mirror families (**Figure 4A**, *t*-test, *P* < 0.0001). This apparently suggested that full-scale coverage indeed confers some protection against the disease. However, this result might be confounded by the genetic background of the groups, since fully scaled families were more closely related to the feral strain.

The BC_1_ families are on average as resistant as the F_1_ families, and furthermore, each family contains both fully scaled and mirror sibs, allowing disentangling the confounding effect of the genetic background. Therefore, fully scaled and mirror sibs were referred to as separate groups in a series of comparisons using the survival data of BC_1_ families only. Unlike before, considering the means of all BC_1_ families together, fully scaled and mirror fish did not differ significantly (*t*-test, *P* = 0.9037, **Figure 4B**). Since it appears that CyHV-3 resistance might be a quantitative trait, testing the means of all families might not be sufficient and it could still be that scale pattern, as one out of possibly many factors affecting the trait, has a small effect on survival that will be significant in some families but not in others. However, among the 27 BC_1_ families tested, not even one family showed a significant difference in survival levels between scaled and mirror siblings (**Figure 4B**, χ^2^ tests, *P* > 0.05).

Finally, “mortality patterns” were compared between scaled and mirror phenotypes in the BC_1_ families. Although mortality levels did not differ significantly, there was a trend for differences in “mortality pattern” (*t*-test, *P* = 0.081, **Figure 4C**). On average, it took scaled fish slightly longer to reach 50% mortality than to mirror fish.

### Differences in Viral Loads between Resistant and Susceptible Strains

**FIGURE 5:**
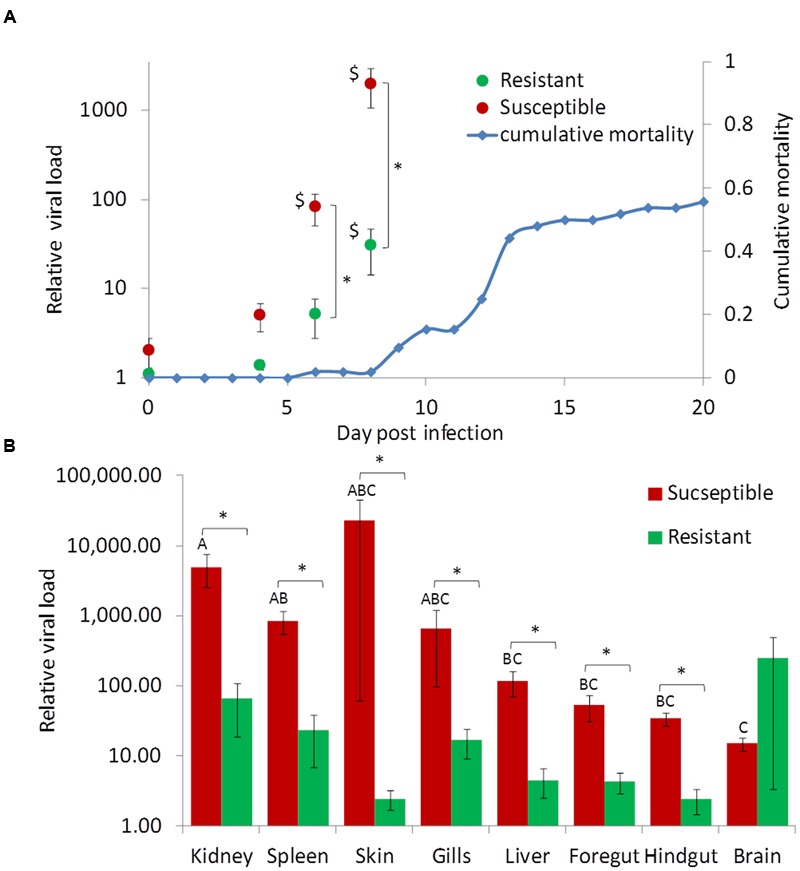
**Viral load in tissues of susceptible and resistant fish during the course of CyHV-3 infection.** For both panels values coming from susceptible and resistant fish are in red and green, respectively. **(A)** Relative mean viral loads in susceptible and resistant fish by day. Blue trend line shows the cumulative mortality in the susceptible control group throughout the challenge experiment. Shown are means and error bars across six replicate fish and three tissues (kidney, spleen, and liver). Significant differences (*t*-test, *P* < 0.05) between susceptible and resistant fish are marked by asterisks and significant differences (Dunnett’s test, *P* < 0.05) from day 0 by $ sign. **(B)** Relative mean viral loads in tissues of resistant and susceptible fish at day 8 post-infection. Shown are means and standard error bars of six replicate fish. Significant differences (*t*-test, *P* < 0.05) between susceptible and resistant fish are marked by asterisks. Red bars not sharing the same capital letters on top are significantly different (Tukey–Kramer test, *P* < 0.05).

Since a protective effect to the outer scale coverage was not detected, we further tested if resistant fish differ from susceptible fish in the levels of viral infection. Fish from two carp families were used, one resistant (“SxS”) and another susceptible (“YxY”), to quantitatively measure the viral DNA loads in several tissues throughout infection by cohabitation done commonly to both groups. As expected, the susceptible group suffered mortalities at the typical time, confirming the development of the disease, while the resistant group did not suffer mortalities at all (**Figure 5A**, see blue trend line).

Having confirmed the development of the disease, viral DNA levels were measured by qPCR throughout infection in susceptible and resistant fish. To look at differences among the four sampling “days,” three “tissues” (spleen, liver, and kidney), in which we found the highest and most consistent virus levels on day 6 and 8 post-infection, were chosen for analyses. The effects of three factors: “day,” “tissue,” and “phenotype” (susceptible/resistant) were tested by a three-way ANOVA model. The triple interaction between the factors was insignificant (*P* = 0.3856), but due to significant pair-wise interactions between “day” and “phenotype” (*P* < 0.0001) and between “day” and “tissue” (*P* = 0.0128), the effect of one factor was tested separately in each level of the other factor.

For susceptible fish, virus levels were insignificantly elevated at day 4, but at days 6 and 8 post-infection virus levels were significantly greater compared to day 0 (Dunnett’s test, *P* < 0.05). For resistant fish, virus levels raised as well but later and to lesser levels. Only at day 8 was there a significant difference from day 0 (Dunnett’s test, *P* < 0.05). In the other test direction, on days 6 and 8, the differences in virus levels between resistant and susceptible fish were significant (*t*-test, *P* < 0.0001 for both days). Notably, the significantly increased virus levels in susceptible fish (days 6 and 8) predated their mortalities, which started only after day 8 when the virus levels were over three orders of magnitude greater than at day 0 (**Figure 5A**). The above analysis was performed on mean values from all three tissues, but due to the interaction between “tissue” and “day” these findings were also verified by analyzing each tissue separately in the same manner. In spleen and kidney the results repeated themselves, while in liver a similar trend was observed but the difference between susceptible and resistant fish was significant only at day 8 (data not shown).

Next, the distribution of viral DNA in fish tissues was examined. Day 8 was chosen for the analysis as it represented fish in a progressive infection stage. The effects of phenotype and tissue were tested by a two-way ANOVA. The effect of the interaction was close to being significant (*P* = 0.0587) and thus, the effect of each factor was tested separately. In line with the average difference (**Figure 5A**), also for each tissue except for the brain, viral DNA levels were significantly greater in susceptible fish (**Figure 5B**; *t*-tests, *P* < 0.05). Most notably, greater levels were found in kidney and spleen whereas lesser in brain (Tukey–Kramer test, *P* < 0.05, **Figure 5B**). In the skin and gills, mean infection levels were high, but so was the variation among replicates, leaving the differences between these and other tissues insignificant. Furthermore, only in susceptible fish were there significant differences between tissues, confirming that infection in different body parts of susceptible fish was faster and to greater levels than in resistant fish.

## Discussion

Outbreaks of infectious diseases in aquaculture inflict tremendous damage to farmers and significantly trouble food security. Since controlling such outbreaks is harder in aquaculture than in land animals farming, preventative solutions for sustainable aquaculture are even more needed. Although it takes considerable efforts and longer time to develop, fish strains resistant to diseases are one desirable and sustainable solution to the problem (Ødegård et al., 2011; Gjedrem et al., 2012; Stear et al., 2012; Bishop and Woolliams, 2014). A prime example for one such successful development and the impact of this achievement on aquaculture comes from Atlantic salmon (*Salmo salar*), where strains with resistance to IPNV (infectious pancreatic necrosis virus) are now routinely used by significant sections of this industry (Houston et al., 2010; Yáñez et al., 2014; Moen et al., 2015). Finding that the trait variation contains a heritable genetic component is a prerequisite for breeding resistant fish strains. Therefore, with the aim in mind to develop CyHV-3 resistant carp strains, in this study the first steps to characterize and understand CyHV-3 resistance were made.

Considerable variation in survival was observed at two levels. One was variation between strains; most notably between the means of the feral (“S”) and the cultured strains (“Y,” “D,” “DxY,” and “Koi”). Variation in survival levels among different carp strains following CyHV-3 infection has been reported before (Shapira et al., 2005; Zak et al., 2007; Dixon et al., 2009; Rakus et al., 2009; Ødegård et al., 2010; Piačková et al., 2013; Ito et al., 2014). Some previous studies, but not all, shared our first observation, namely resistance of the feral strain in contrast to susceptibility of the cultured strains. Seemingly, this observation puts breeding for CyHV-3 resistance in a question mark since the feral strain is not favorable for aquaculture and thus, further crossbreeding generations to cultured strains were required to prove this natural resistance source could be utilized for breeding.

The other level was variation among means of different families; most notably for F_1_ (“SxY” and “SxD”) and BC_1_ [“Yx(SxY)” and “Dx(SxD)”] families. Also variation in survival levels among families of the same genetic background, had been reported before (Dixon et al., 2009; Rakus et al., 2009; Ødegård et al., 2010; Piačková et al., 2013). Our results, however, showed that in F_1_ between the resistant feral and the susceptible cultured strains, the trait variation among families was larger than for each of the parental lines. Moreover, as far as we could tell, this study was first to measure variation in CyHV-3 resistance in BC_1_ families and to show that this variation was not necessarily dominant and it was heritable beyond the F_1_ hybrid generation. Thus, such considerable variation in F_1_ serves as a good starting point for an effective family-based selection of resistant strains, and the successful inheritance of this resistance by BC_1_ families proves the potential of this strategy.

In our study, CyHV-3 resistance was found in a feral strain, which is unsuitable for farming. As important as CyHV-3 disease resistance is, introgression of this trait from the feral strain to cultured strains could come with a cost in other desirable traits that cultured strains were already bred for. Therefore, in fish, much like in land farm animals, we often see that selection programs to include a new trait avoid crossing improved breeds to wild/feral strains and instead, focus their selection on trait variation existing within the cultured breeds. Accordingly, more than one example exists of selection programs to improve disease resistance in fish that involve cultured strains only (Price, 1985; Henryon et al., 2002; Vallejo et al., 2010; Wiens et al., 2013; Yáñez et al., 2014). In common carp, most trait variation in CyHV-3 resistance is found between cultured and feral or feral-related strains (Shapira et al., 2005; Dixon et al., 2009; Ødegård et al., 2010; Piačková et al., 2013). Some carp strains cultured in the Czech Republic have better resistance to CyHV-3. However, most of these strains are scaly and genetically related to the feral “S” strain (Piačková et al., 2013), therefore it is probable that these lines already contain introgressions from the feral “S” strain or other feral strains which contributed to their resistance. In contradiction to the low trait variation found in cultured strains in this study, there are reports of a few European cultured strains with greater CyHV-3 resistance trait variation (Ødegård et al., 2010; Piačková et al., 2013). It is yet to be tested if resistance can be improved in these strains through a selection program. However, given high heritability estimates reported for this trait (Ødegård et al., 2010) it appears possible.

In our study, the introgression approach from the feral to cultured strains appears to be successful, however, it is yet to be seen how BC_1_ and more advanced BC generations will perform in other desirable traits. So far, there are a few good indications also with respect to other traits. First, with respect to reduced scale pattern (mirror phenotype), which in several countries is considered a desirable trait. Since it is a simple trait (Kirpichnikov, 1981; Rohner et al., 2009) differentiating the feral “S” from the cultured “D” and “Y” strains, already at the BC_1_ generation, half of each progeny were mirror fish that resembled the cultured strains in this respect and possibly in others too. In addition, the BC_1_ analyses indicated that the scale coverage pattern was not correlated with CyHV-3 resistance and therefore, it should be possible to obtain resistant mirror strains using our strategy. Secondly, with respect to genetic variability and inbreeding, the parental stocks of “Y” and “D” strains are kept as closed inbred populations since the 1970s and consequently they present reduced genetic variation and possibly also some degree of inbreeding depression (Moav et al., 1975; Kincaid, 1983; David et al., 2001, 2007). Survival levels after CyHV-3 infection could also be looked at as an example trait from which we can learn on variation among our strains. In F_1_ and BC_1_ generations more variation in survival levels among families was observed compared to the parental strains. Crossing of these inbred cultured strains with the genetically different feral strain increased genetic variation and probably reduced inbreeding depression. Thus, despite the potential risk, breeding using an introgression approach looks promising for improving disease resistance as well as for reducing inbreeding levels and consequentially has the potential to improve other traits too.

In this study, we also started looking at what the mechanism of CyHV-3 resistance could be. There is a distinction between tolerance, which is defined as the capacity of the host to withstand the damages inflicted by the pathogen, and resistance, which is defined as the capacity of the host to control the pathogen infection (Råberg et al., 2007; Bishop and Woolliams, 2010; Doeschl-Wilson et al., 2012; Glass, 2012). Control over pathogen infection could be obtained by blocking the pathogen from infecting internal organs or, if the fish was already infected, by limiting the pathogen load in the tissues. In the case of IPNV resistance in Atlantic salmon, there was strong evidence that a major part of the resistance was due to blocking the pathogen from infecting resistant fish (Moen et al., 2015). Our results for CyHV-3 infection justify defining our observations as resistance/susceptibility rather than tolerance. In our experiments, at day 8 post-infection by cohabitation, significantly greater viral DNA levels were found in several tissues of susceptible fish compared to resistant ones. This observation clearly suggested that also resistant fish were infected, but unlike susceptible fish, were capable of controlling the level of infection. Finding differences in viral DNA levels at day 8 among different tissues in susceptible but not in resistant fish is another indication for the capacity of resistant fish to control the viral spread inside their bodies.

While more studies characterized CyHV-3 infection and carp responses to infection (Adamek et al., 2014), not many have identified genetic differences that might contribute to resistance. One study reported that differences in survival levels were associated with genetic differences in *Cyca-DAB1-like* genotypes (Rakus et al., 2009), one of the major histocompatibility (MHC) class II B genes of the common carp. Often, MHC genes were proposed to be good candidates for contributing to disease resistance and this finding in carp is in line with similar findings in other fish and land animals (Price, 1985; Chevassus and Dorson, 1990; Xu et al., 2010; Du et al., 2011; Glass, 2012; Bishop and Woolliams, 2014; Das and Sahoo, 2014; Yáñez et al., 2014). Another group of genes, the innate immune response genes, are good candidates for contributing to disease resistance. In a collaborative study, we have previously identified polymorphisms in several innate immune response genes from carp (Kongchum et al., 2010) and found that different genotypes of *IL10a*, one of two *Interleukin 10* gene copies found in common carp, were associated with CyHV-3 resistance (Kongchum et al., 2011). Given the variability in survival levels we found among F_1_ and BC_1_ families in both genetic backgrounds (crosses of “S” to “Y” or “D”), and given the strong heritable component we found for this trait, our previous and current findings suggested that CyHV-3 resistance is a multigenic trait. Several examples exist where disease resistance seems to be conditioned by a single or major gene (Fuji et al., 2006; Moen et al., 2009; Gheyas et al., 2010; Houston et al., 2010; Baerwald et al., 2011; Verrier et al., 2013). On one hand, traditional and marker-assisted breeding to improve disease resistance might be straighter forward when most trait variability is explained by a single locus. However, as long as the additive genetic component is significant, as it has been estimated for several disease resistances (Ødegård et al., 2011; Stear et al., 2012; Das and Sahoo, 2014; Yáñez et al., 2014; Palti et al., 2015) and as shown here for CyHV-3 resistance, breeding for improved strains is not only important but also feasible.

In conclusion, in our study we identified the feral “S” strain as a source for introgression of CyHV-3 resistance into susceptible cultured strains and demonstrated the feasibility and advantages of this strategy up to generation BC_1_. Considering the mean survival levels of different families as the measure for resistance, both F_1_ and BC_1_ families presented large trait variation, which is a good starting point for family based selection of resistant broodstock lines. Since in these genetic backgrounds, CyHV-3 resistance has a significant additive genetic component, this selective breeding strategy should work out fine and allow obtaining relatively quickly lines with high level of resistance (at least in laboratory conditions). Furthermore, as CyHV-3 resistance is likely to be a multigenic trait, having both resistant and susceptible families on a similar genetic background (F_1_ or BC_1_) rather than in genetically different strains (feral vs. cultured) should expedite further studying the mechanism of resistance and moreover, the genetic basis of this trait.

## Author Contributions

RT-L, GH, and LD conceived and designed the experiments, RT-L, EA, and LD executed the experiments, RT-L, EA, and LD summarized the results and analyzed the data, GH and LD provided materials and supervised the research, RT-L, GH, and LD wrote the manuscript.

## Conflict of Interest Statement

The authors declare that the research was conducted in the absence of any commercial or financial relationships that could be construed as a potential conflict of interest.
